# The risk analysis of perioperative complications of cementless hip arthroplasty in octogenarians

**DOI:** 10.1007/s00402-022-04575-2

**Published:** 2022-08-26

**Authors:** Julian Koettnitz, Justus Jäcker, Filippo Migliorini, Michael Trost, Christian Dominik Peterlein, Christian Götze

**Affiliations:** 1 Department of Orthopaedic Surgery, Auguste-Viktoria Clinic, Ruhr University Bochum, 32545 Bad Oeynhausen, Germany; 2 Department of Orthopaedics and Trauma Surgery, University Clinic Aachen, RWTH Aachen University Clinic, 52064 Aachen, Germany; 3 Department of Orthopaedic and Trauma Surgery, RWTH Aachen University Hospital, Pauwelsstraße 31, 52074 Aachen, Germany; 4 Department of Orthopaedics and Traumatology, St. Josef-Hospital, Ruhr University Bochum, Bochum, Germany

**Keywords:** Octogenarians, Systemic complications, Cementless hip arthroplasty, Blood transfusion, Hospitalisation

## Abstract

**Introduction:**

Hip arthroplasty is exposed to demographic change as patients age. Analysis of risk factors for surgical treatment decisions in the group of ≥ 80-year-old patients is crucial. Healthcare systems in developed countries are being tested medically and financially by the ageing population. Therefore, this study analysed the perioperative complications of cementless primary hip arthroplasty in octogenarians and compared them with patients aged ≤ 60 years.

**Methods:**

A retrospective data analysis of the year 2017 was done in a maximum care hospital of General Orthopaedic Surgery. Patients aged ≥ 80 years or ≤ 60 years with primary cementless hip arthroplasty were included. The outcome of interest was surgery-related and systemic complications, the development of haemoglobin and the incidence of blood transfusion after cementless primary hip arthroplasty in octogenarians during the hospitalisation and the follow-up treatment. Chi-square tests and Fischer's exact test were used for nominal variables. The two-factorial variance analysis-mixed model was used for Hb analyses and the Welch test for group comparison for metric parameters.

**Results:**

There was a significantly increased incidence of systemic complications during hospitalisation in the ≥ 80-year-old patients (phi 0.26; Std. Ri − 0.8 (A), 2.2 (B); *p* = 0.007), as well as a significantly increased rate of blood transfusions (phi 0.403; Std. Ri − 1.3 (A), 3.2 (B); *p* = < 0.001). No clustered pre-existing conditions in the ≥ 80-year-old patients pointed out a significant association with the incidence of systemic complications. Surgery-related complications showed no significant difference during hospitalisation and follow-up treatment.

**Conclusion:**

The study reveals that primary cementless hip prosthesis implantation is a safe procedure without increased incidence of surgery-related complications. Increased attention should be paid to interdisciplinary preoperative optimisation (adjustment of blood pressure, blood transfusions, if necessary, safe exclusion of urinary tract infections) and postoperative care of octogenarians (tight laboratory examinations, geriatric co-attendance).

## Introduction

The overall age of the population in industrial countries is increasing, and therefore, the prevalence of end-stage degenerative joint diseases [1]. Therefore, the number of arthroplasties performed on octogenarians is expected to increase [2]. Total hip arthroplasty (THA) aims to restore the function and biomechanics of the hip joint [3]. However, such a procedure in octogenarians may be associated with enhanced complications. Reduced performance status and lower preoperative haemoglobin have been described as risk factors [4]. In octogenarians, an increased rate of cognitive dysfunction and deep vein thrombosis have been demonstrated. In addition, cardiovascular and urogenital complications were also reported. Octogenarians seem to be more susceptible to anaesthesia- and surgery-associated mortality in major surgeries [5–7]. Some studies showed a slow survival probability for endoprosthesis in octogenarians. [8, 9]. The management of complications in elderly patients can be lengthy. The possibility of potentiating events over the inpatient treatment can lead to responsive medicine. To our knowledge, no other retrospective study has directly compared perioperative complications between a particularly young versus a particularly old patient group. Furthermore, no other study combined local, surgical, and systemic complications, as well as blood transfusion frequencies.

This study was initiated to identify possible common complications of octogenarians undergoing primary hip arthroplasty to take preventive measures in clinical practice. Possible prolonged inpatient hospital stays due to infections; mobility disorders or surgical complications should thus be reduced. Therefore, summarising of the main complications of surgical and systemic nature was done. Subsequently, the complications were compared with age-typical pre-existing diseases. Local, surgical, and systemic complications were recorded. Furthermore, the frequency of blood transfusions was investigated. Afterwards, all data was compared between young patients under the age of 60 and octogenarians. It was hypothesised that perioperative complications, as well as blood transfusions, were more common in octogenarians.

## Materials and methods

### Patient recruitment

The present study was conducted according to the Strengthening the Reporting of Observational Studies in Epidemiology (STROBE) statement [10]. A retrospective data analysis of the year 2017 was done in the Department of Orthopaedic Surgery of the RUB university hospital Auguste-Viktoria-Klinik. 356 primary cementless hip arthroplasties were done in 2017. All data came from a house intern archiving program and were available in electronic form. Access to the surgical progression, duration of stay in the hospital and post-hospital documentation for patients was possible through the program. Data collection was done over 5 months from November 2018 to March 2019.

### Eligibility criteria

The inclusion criteria were (1) age 80 years and older or 60 years and younger, (2) cementless hip arthroplasty of the cup and stem, (3) patients with primary elective hip arthroplasties, (4) standard minimal invasive anterolateral approach, and (5) implantation of the Allofit-System and femoral Fitmore^®^ stem (Fa. Zimmer Biomet Freiburg GE). The exclusion criteria were (1) ages between 61 and 79, (2) revision hip arthroplasties, (3) cemented hip arthroplasties, (4) different approach than anterolateral, (5) different type of cup and stem, (6) intraoperative change of method, stem and cup, and (7) incorrect documented patient files pre-, intra-, postoperative and in the follow-up treated. The data of the patients were collected chronologically in the excel spreadsheet.

### Surgical procedure

Surgical procedures were performed by 12 surgeons. Anterolateral approach, Fitmore^®^ stem as well as pre-and postoperative management was done for all patients.

The surgical procedure was performed in a lateral position using a minimally invasive technique. An incision was made from the greater trochanter in the direction of the SIAS (spina iliaca anterior superior*)*. After cutting the muscle fascia, the gluteus medius muscle was retracted to provide a clear view of the joint capsule. After the preparation of the capsule, the two-stage osteotomy was performed, first near the femoral head and then at the level of the trochanteric fossa. After removal of the head, preparation of the acetabulum was performed with a press fit cup implantation. Turning towards the femur: here, the medullary canal is displaced by utilising a fine rasp. After implantation of the trial stem and the head, trial implantation was performed. If the conditions were stable and the leg length was correct, the final prosthesis was implanted. In the rehabilitation phase, immediate full mobilisation without restrictions was performed with the help of physiotherapy.

### Data assessment

Categorisation of the patient cohort into two age groups (A < 60 years; B ≥ 80 years) was done, based on a monocentric, retrospective case–control study. The period of documentation in the follow-up treatment for group A was about 151 ± 147 days, for group B about 88 ± 84 days.

### Outcome of interest

Preoperative data: the age, the gender, the BMI, the length of the hospital stay, the duration of the surgery, the operating doctor, pre-existing conditions, visual analogic scale and nicotine use were recorded. Intraoperative data: the size and type of cup, the inlay and stem were collected. Postoperative data: haemoglobin, surgery-related complications like open and closed reposition, periprosthetic fracture, dislocation, leg length difference, delayed wound healing, nerve lesion and systemic complications (urinary infection, embolism, thrombosis, infarction, myocardial infarction, arrhythmia, pneumonia, pulmonary oedema, renal insufficiency, abdominal complications), as well as the influence on an increased blood transfusion incidence, were examined. In the follow-up treatment, documented surgical revisions, interventions and infections were collected. Failure was defined as an intraoperative change of the implant, a different approach than anterolateral, mortality during hospitalisation and follow-up treatment. Pre-existing conditions that occurred more frequently in old age were investigated in addition with the systemic complications. Data were assessed after the cases were closed and after documented follow-up treatment.

### Statistical analysis

Data were collected using Microsoft^®^ Excel^®^ for Office 365 and statistical analysis was performed using IBM^®^ SPSS^®^ 28 (Armonk, New York, USA). Continuous variables were described by median and quartiles, means and standard deviations. Absolute and relative frequencies were used to represent categorical variables. Nominal variables were analysed by Chi-square tests and by Fischer’s exact test. The effect size was measured using phi (0.10 small effect; 0.3 middle effect; 0.5 high effect) and standardised residuals (> 2, regardless of minus or plus, shows a meaningful effect). The two-factorial variance analysis-mixed model was used to compare group differences. The effect size was determined by squared Eta (0.01 small effect; 0.06 middle effect; 0.14 high effect). The *p* values reported should be understood as descriptive measures. The Welch test for independent samples was used for group comparisons for metric parameters. The effect size was determined by Cohen’s *d* (0.20 small effect; 0.50 middle effect; 0.80 high effect). The significance level was set two sided with *α* = 0.05.

## Results

### Enrolment process

**Fig. 1:**
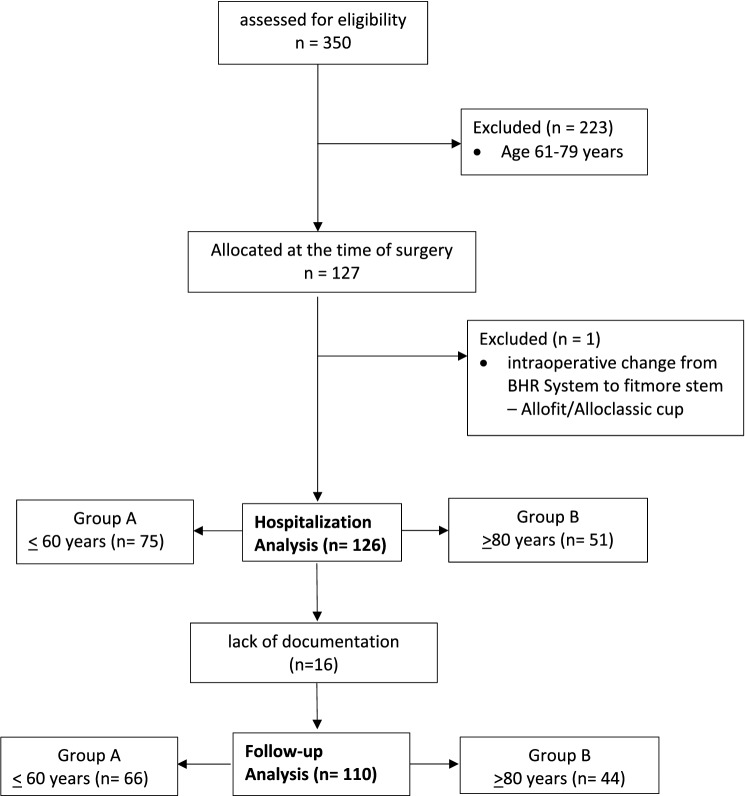
Flowchart of the enrolment process

A total of 350 patients were initially screened. 223 of the patients were between 61 and 79 years old (*n* = 223). 127 patients were suitable for the study. 51 patients were 80 years and older and compared with 76 patients 60 years and younger. In one case, a BHR^®^ system was initially implanted. Intraoperatively, the surgeon decided to switch to a Fitmore^®^ stem. The prosthesis was treated as a revision prosthesis and, therefore, dropped from the evaluation (*n* = 1). 126 patients were left for the investigation. 51 patients were 80 years and older and 75 patients were 60 years and younger (Fig. 1). All patients underwent primary cementless hip arthroplasty. In the follow-up treatment some patients could not be considered due to missing documentation (*n* = 15). For the follow-up analysis in Group A ≤ 60-year-old patients *n* = 66 were left, and in Group B ≥ 80-year-old patients *n* = 44 were left.

### Demographic data

We analysed the data of 126 patients during hospitalisation and 110 patients during the follow-up treatment. Overall, 56.34% (71 of 126) were women, and 43.66% (55 of 126) were men. The mean age in group A was 51.8 years and in group B 83.3 years. The mean BMI in group A was 29.37 kg/m^2^ and in group B 26.62 kg/m^2^. The mean ASA-Score (American Society of Anaesthesiologists) in group A was 1.70 and in group B 2.45. The mean hospitalisation was 9 days for group A and 10 days for group B. Hospital stay between age groups differed significantly with longer stay for patients 80 years and older (*df* 82,407; *d* = 0.531; *p* = 0.008). The main follow-up treatment of group A was 170 days and 100 days for group B (Table 1).

**Table 1:** Demographic data

Endpoint	Group A (*n* = 75)	Group B (*n* = 51)
Follow-up (days)	170 ± 22.42	100 ± 14.55
Age	51.8 ± 5.80	83.3 ± 2.63
Gender (female)	52%	62.74%
BMI (kg/m^2^)	29.37 ± 5.73	26.62 ± 3.68
ASA-Score	1.70 ± 0.60	2.45 ± 0.53
Hospitalisation (d)	9.03 ± 1.94	10.25 ± 2.77

*ASA-Score* American Society of Anaesthesiologists, *BMI* body mass index

### Systemic and surgical complications

#### Hospitalisation

Table 2 shows the occurrence of systemic and surgery-related complications during the hospitalisation.

**Table 2:** Systemic and surgical complications during hospitalisation

Systemic complications	Surgery-associated complications
**Urogenital (*n* = 7):** Urinary tract infection	**Revision occurred (*n*** **= 5):** Periprosthetic fracture (*n* = 3) → Stem change, cerclages, head and inlay change Open wound more than 7 days (*n* = 1) → Hematoma debridement Leg length difference > 2 cm (*n* = 1) → Stem change
**Cardiac/vascular (*n*** **= 3):** Myocardial infarction (*n* = 1) Cardiac arrhythmias (*n* = 2)	
**Renal (*n*** **= 1):** Acute renal insufficiency	**No revision occurred (*n*** **= 3):** Irritation in sciatica (*n* = 1) → Electrotherapy, physiotherapy Open wound more than 7 days (*n* = 1) → Vacuum dressing, close wound controls Luxation (*n* = 1) → Closed reposition

#### Systemic complications

In total, systemic complications occurred in 11 patients (8.73%) during their hospital stay (Table 3). With 9 patients (17.6%), group B showed the highest incidence of complications and showed significant differences compared to group A 2.6% (n = 2) (p = 0.007).

**Table 3:** Systemic complications (urinary infection, myocardial infarction, cardiac arrhythmia, renal insufficiency)

Endpoint	Group A (*n* = 75)	Group B (*n* = 51)	Phi	Std. Ri	*p*
Systemic complications	2.6% (2/75)	17.6% (9/51)	0.26	− 1.8 (A), 2.2 (B)	0.007

#### Surgery-associated complications

There was no statistically significant difference of complications in need of revision between group A and B (Tables 4, 5). In group A, there were 2.6% (*n* = 2) and in group B 5.9% (*n* = 3). Likewise, there was no statistically significant difference of complications without the need of revision between group A and B. In group A, there were 4% (*n* = 3) and in group B 0% (*n* = 0).

**Table 4:** Hospitalisation (surgical interventions: stem change, cerclages, head inlay change, hematoma debridement, closed reposition)

Endpoint	Group A (*n* = 75)	Group B (*n* = 51)	Phi	Std. Ri	*p*
Revision occurred	2.6% (2/75)	5.9% (3/51)	0.081	− 0.6, 0.7	0.394

**Table 5:** Hospitalisation (local arrangements: electrotherapy, physiotherapy, vacuum dressing, close wound controls)

Endpoint	Group A (*n* = 75)	Group B (*n* = 51)	Phi	Std.Ri	*p*
No revision occurred	4% (3/75)	0% (0/51)	0.129	0.9, − 1.2	0.217

### Follow-up

Table 6 shows the occurrence of surgery-related complications during follow-up treatment.

**Table 6:** Surgical complications during the follow-up

Surgery-associated complications (follow-up)	
Revision occurred (*n* = 5)	No revision occurred (*n* = 4)
Luxation (*n* = 1) → Head and inlay change, cup change Periprosthetic infection (*n* = 1) → Two-stage exchange operation of the prosthesis Dislocation of trochanter major (*n* = 1) → Re-fixation of the greater trochanter Persistent deep and superficial wound seroma (*n* = 1) → Head and inlay change (*n* = 1)	Femoral nerve lesion (*n* = 1) → Continuation of physiotherapy Luxation (*n* = 1) → Closed reposition Persistent superficial wound seroma (*n* = 1) → Puncture, oral antibiotics Periarticular ossification (*n* = 1) → Intensified physiotherapy

#### Surgery-associated complications

#### Surgery-associated complications

In total, surgery-associated complications during the follow-up treatment occurred with 3.0% (*n* = 2) in Group A and 6.8% (*n* = 3) in group B (Tables 7, 8). There was no statistically difference between the groups. Likewise, there was no statistically significant difference of complications without the need of revision between group A and B. In group A, there were 1.5% (*n* = 1) and in group B 6.8% (*n* = 3).

**Table 7:** Follow-up (surgical interventions: head and inlay change, cup change, two-stage exchange operation, re-fixation greater trochanter)

Endpoint	Group A (*n* = 66)	Group B (*n* = 44)	Phi	Std. Ri	*p*
Revision occurred	3.0% (2/66)	4.5% (3/44)	0.040	− 0.3, 0.3	1

**Table 8:** Follow-up (local arrangements: physiotherapy, closed reposition, punction, oral antibiotics)

Endpoint	Group A (*n* = 66)	Group B (*n* = 44)	Phi	Std. Ri	*p*
No revision occurred	1.5% (1/66)	6.8% (3/44)	0.136	− 9, 1.1	0.300

### Preconditions and systemic complications

Arterial hypertension occurred in 76.47% (*n* = 39) of group B and 37.33% (*n* = 28) in group A. No statistical association between arterial hypertension and the occurrence of systemic complications was found (*p* = 0.06). Antithrombotic medication, which was taken in 52.94% (*n* = 27) of group B compared to group A 1.33% (*n* = 1) did not point out to be a significant factor for systemic complications (*p* = 0.772). ASA-Score > 3, which appeared in 49.01% (*n* = 25) in group B and 6.6% (*n* = 5) in group A did not influence systemic complications (*p* = 0.291). Likewise, no statistical correlation to systemic complications could be found in renal insufficiency, which was only detected in group B at 21.56% (*n* = 11). Arrhythmia was not associated with a risk of systemic complications (*p* = 0.226). In 31.37% (*n* = 16) of the patients in group B and 10.66% (*n* = 8) in group A, arrhythmia was described. Furthermore, a statistical association between the BMI > 30 and nicotine consumption with systemic complications was investigated (Table 9). Both pre-existing conditions occurred more frequently in group A (BMI > 30 = 41.33%; *n* = 31; NC = 28%; *n* = 21) than group B (BMI > 30 = 15.68%; *n* = 8; NC = 3.92%; *n* = 2). There was no statistical association between systemic complication and the BMI > 30 or nicotine consumption (BMI > 30 *p* = 1; NC *p* = 0.687).

**Table 9:** Pre-conditions and association to systemic complications

Endpoint	Group A (*n* = 75)	Group B (*n* = 51)	Phi	Std. Ri	*p*
Arterial hypertension	37.33% (28/75)	76.47% (39/51)	0.17	1.3, − 1.4	0.06
Antithrombotic medication	1.33% (1/75)	52.94% (27/51)	0.02	− 0.1, 0.3	0.772
ASA > 3	6.6% (5/75)	49.01% (25/51)	0.091	0.9, 0.5	0.291
Renal insufficiency	0%	21.56% (11/51)	0.10	− 0.3, 1.1	0.246
Arrhythmia	31.37% (16/75)	10.66% (8/51)	0.128	− 0.6, 1.2	0.226
BMI > 30	41.33% (31/75)	15.68% (8/51)	− 0.025	0.1, − 0.2	1
Nicotine consumption	28% (21/75)	3.92% (2/51)	− 0.073	0.3, − 0.7	0.687

### Development of haemoglobin and transfusion

The development of haemoglobin (Hb) within the age groups could be taken from routine laboratory tests. Values before an operation and before the discharge from hospital (day 1, day 7) were compared. Preoperatively, age group B showed a value of 133.73 g/l (SD 12.89) in comparison to group A and 145.10 g/l (SD 11.92) in those under 60 years of age. The Hb drop 1 day after surgery was not significantly different between groups A and B (*p* = 0.321). For measuring the Hb drop between preoperative and Day 7, we analysed group A *n* = 59 and group B *n* = 46, because of missing values for day 7 of 16 patients in group A and 5 patients in group B. The Hb drop from preoperative to day 7 postoperative was not significantly different between groups A and B (*p* = 0.889). According to the WHO Criteria, there were 69.33% of patients in group A and 90,19% of patients in group B with persisting anaemia on day 7 (Table 10). Postoperative transfusion of blood occurred significantly more often in group B (31.4%) than in group A (2.7%) (*p* < 0.001) during hospitalisation.

**Table 10:** Haemoglobin drops preoperative—Day 1 and Day 7 postoperative; blood transfusion postoperative

Endpoint	Group A	Group B	*df*	*η*^2^	*p*
Drop of haemoglobin preOP-Day 1	23.31% (33.65 g/l)	24.74% (35.67 g/l)	1	0.008	0.321
Drop of haemoglobin preOP-Day 7	24.65% (35.78 g/l)	26.47% (35.41 g/l)	1	0.000	0.889

Endpoint	Group A (*n* = 75)	Group B (*n* = 51)	Phi	Std. Ri	*p*
Blood transfusion	2.7% (2/75)	31.4% (16/75)	0.403	− 1.3 (A), 3.2 (B)	0.001

## Discussion

Regarding the current study, a difference in the frequency of systemic complications was observed between the group of patients aged ≤ 60 years and the group of patients aged ≥ 80 years. However, there was no significant difference in the comparison of pre-existing conditions, which are more common in older individuals, to systemic complications. Likewise, no significant difference was found for surgical complications or local complications during hospitalisation and the follow-up treatment. The study of the haemoglobin drop showed no significant difference between the two age groups. A significant difference was found in the rate of blood transfusions required.

In the context of demographic change and increasing demands on the quality of life, geriatric patients place high demands on arthroplasty. Currently, the problems and treatment options in hip arthroplasty in a patient 80 years and older are discussed [11, 12].

Arthroplasty in patients over 75 years of age is performed with both cementless and cemented anchorage. There is a controversial discussion about the failure probability and service life of endoprosthesis in elderly patients. Nanjayan et al. showed a higher incidence of cardiovascular disease and pneumonia for octogenarians after THA. Yohe et al. examined the risk of readmission to hospital and complications within 30 days for patients ≥ 80 years. In their investigation, chronic obstructive pulmonary disease was the only comorbidity, which increased the risk for readmission to the hospital. Overall, patients above the age of 80 showed more major and minor complications after THA. Furthermore, octogenarians have been shown to have an increased risk of urogenital infections, as well as postoperative delirium [13, 14]. Urinary tract infections were also common in our study. Prolonged use of a bladder catheter in older patients could increase the risk compared to younger patients due to possible delayed mobility postoperatively. In addition, certain pre-existing conditions could also be generally well controlled by the general practitioner. Kelly et al. 2022 documented cause-specific revision risks by implantation method, age, and gender. There was a high risk of periprosthetic fractures for female patients ≥ 75 for cementless hip arthroplasty in comparison to hybrid implantation. In contrast, no significant differences in the incidence of complications related to older age were found in other studies [8, 9]. Other investigators showed a lower risk of dislocation in a 5-year follow-up treatment for octogenarians with cementless stem and dual mobility cups [15, 16]. Tsukada et al. demonstrated a 93% survival rate in a 14-years follow-up treatment for cementless hip arthroplasty with ceramic-on-alumina ceramic components [17]. The condition of materials and the combination can also affect the risk of surgery-related complications. In this study, comparatively very few patients showed perioperative surgery-related complications. The reason could be the established standardised implantation technique for primary hip arthroplasty. The surgical routine may create safety during the operation and, therefore, enables a higher quality, also in the training. Other studies showed a prolonged hospitalisation of older patients; however, a generally worse outcome could not be proven [16, 18]. The hospitalisation in this investigation differed significantly between the age groups. Delayed mobility, due to postoperative delirium, which was not investigated in this study, or continued immobilising pain due to other joints could be relevant here. Standardised, structured hospital processes from surgery to discharge management are thus once again proving to be relevant. Despite the investigation of differences between young and old patients, there are several comparisons between octogenarians and nonagenarians undergoing THA and TKA. The outcome between octogenarians and nonagenarians differed in favour of patients younger than 90 years. Postoperative complications and mortality were increased in the older comparison group in some studies [6, 19]. In this study, octogenarians showed a higher ASA-Score (2.45), but there was no statistical significance to systemic complications (ASA-Score > 3; *p* = 0.291). This is in line with McConaghy et al. who described that the ASA-Score cannot predict minor and major complications [20]. Several studies described a relationship between age and cardiac complications [21–23]. Waterman et al. and Belmont et al. reported increased odds of cardiac complications for patients ≥ 80 years (OR 4.39; 95% CI 2.29–6.61; *p* < 0.001 and OR 3.72; CI 95% 1.53–9.06; *p* = 0.0001) [24, 25]. Dugdale et al. showed hospital mortality of 0.18% and a postoperative infection rate of 0.04% for 147.281 patients in a nationwide research. 24% of the patients needed a blood transfusion. With regards to blood transfusions, current data analyses show that patients over 80 years of age are affected significantly more frequently than younger patients [14, 26, 27]. On the other hand, Hourly et al. showed 0% blood transfusion rate after primary total hip arthroplasty for 153 patients aged about 77 years during hospitalisation. In this investigation blood transfusion in the group of ≥ 80 patients were more common. As the haemoglobin drop was similar in both groups, one explanation could be the more frequent occurrence of clinical symptoms in patients over 80 years of age. Furthermore, an age-related delayed healing tendency compared to younger patients could be the cause. Maybe, a computer-generated system, which could give a fast and individual risk analysis of patients could help to prevent perioperative complications. Kunze et al. were able to build a test system, which had a good predictive capability of about 0.88 (AUC = area under the curve) [28]. However, further investigations with a higher number of patients and further testing of digital intelligence should be performed.

This study has several limitations. The limited number of patients included in the present investigation may limit the ability to detect uncommon complications. The lack of 16 patients in the follow-up treatment was not possible to close. For some patients, the availability of a telephone was not given and for others, we did not receive any feedback. Similar studies involving a larger population and longer follow-up treatments are required. Only the data for 2017 were evaluated. Some values of the visual analogue scale or laboratory results were missing. In 2017, the assessment of mobility and activity pre- and postoperatively was not performed uniformly by means of scores and thus could not be evaluated adequately. Due to the statistical evaluation of mainly nominal scaled values, multiple testing was not feasible in a meaningful way. There was no uniform scoring system for the admission to the hospital or the discharge from hospital of patients. The number of different operating doctors with different operating times could affect the results. Furthermore, no correction for multiple testing was made in the statistical analysis. The calculated *p* values only serve to describe the patient groups in detail and to show possible correlations. Some correlations showed a small to middle effect strength. The relatively small study group of ≥ 80-year-olds with only 51 patients bears the risks of not realistically representing possible differences between the groups due to the missing occurrence of events, as well as falsely representing differences that result solely from the random recording of individual events. Differences between complications and identified risk factors should, therefore, be validated on a larger sample. With the continued paucity of data on the internal medicine outcome of elderly patients after cementless metaphysical fixed prostheses, this work provided a valuable addition to the existing literature. The investigation of the long-term survival of these prostheses in elderly patients with poor bone quality is reserved for other studies.

## Conclusion

Overall, the age group of ≥ 80 years carried a risk for the occurrence of internal (systemic) complications in the inpatient treatment compared to the other age group. There was no difference for surgical or local complications. Preconditions, which occur more often in elderly patients, did not report relation to systemic complications. There was no significant difference in the drop of haemoglobin. In this investigation, blood transfusion rates were more often in the group of ≥ 80-year-olds.
