# Correction to: The risk analysis of perioperative complications of cementless hip arthroplasty in octogenarians

**DOI:** 10.1007/s00402-022-04622-y

**Published:** 2022-09-23

**Authors:** Julian Koettnitz, Justus Jäcker, Filippo Migliorini, Matthias Trost, Christian Dominik Peterlein, Christian Götze

**Affiliations:** 1 Department of Orthopaedic Surgery, Auguste-Viktoria Clinic, Ruhr University Bochum, 32545 Bad Oeynhausen, Germany; 2 Department of Orthopaedics and Trauma Surgery, University Clinic Aachen, RWTH Aachen University Clinic, 52064 Aachen, Germany; 3 Department of Orthopaedic and Trauma Surgery, RWTH Aachen University Hospital, Pauwelsstraße 31, 52074 Aachen, Germany; 4 Department of Orthopaedics and Traumatology, St. Josef-Hospital, Ruhr University Bochum, Bochum, Germany

## Correction to: Archives of Orthopaedic and Trauma Surgery 10.1007/s00402-022-04575-2

The original version of this article unfortunately contained a mistake. Author name Matthias Trost was incorrectly written as Michael Trost.

